# W-boson production in TMD factorization

**DOI:** 10.1140/epjc/s10052-021-09202-9

**Published:** 2021-05-13

**Authors:** Daniel Gutierrez-Reyes, Sergio Leal-Gomez, Ignazio Scimemi

**Affiliations:** 1 Departamento de Física Teórica and IPARCOS, Universidad Complutense de Madrid (UCM), Plaza Ciencias 1, 28040 Madrid, Spain; 2 Faculty of Physics, Wien Universität, Boltzmanngasse 5, 1090 Vienna, Austria

## Abstract

At hadron colliders, the differential cross section for *W* production can be factorized and it is sensitive transverse momentum dependent distributions (TMD) for low boson transverse momentum. While, often, the corresponding non-perturbative QCD contributions are extrapolated from *Z* boson production, here we use an existing extraction (based on the code Artemide) of TMD which includes data coming from Drell–Yan and semi-inclusive deep inelastic scattering, to provide checks and predictions for the *W* case. Including fiducial cuts with different configurations and kinematical power corrections, we consider transverse momentum dependent cross sections within several intervals of the vector boson transverse mass. We perform the same study for the $p_T^{W^-}/p_T^{W^+}$ and $p_T^Z/p_T^W$ distributions. We compare our predictions with recent extractions of these quantities at ATLAS and CMS and results from TeVatron. The results encourage a broader experimental and phenomenological work, and a deeper study of TMD for the *W* case.

## Introduction

Vector boson production is very relevant at hadron colliders and its measurement has achieved an increasing precision in the latest years [1–25]. The experimental results have allowed the extraction of important Standard Model quantities like collinear parton densities and the mass of the *W*-boson [26–29]. The precise measurements of *W* cross section and similar observables need a good determination of QCD non-perturbative inputs which can be partially extracted from neutral boson mediated processes. Because we are interested in transverse momentum dependent observables it is mandatory to consider the extraction of transverse momentum dependent parton distribution functions (TMD) that have used the data of the neutral bosons at low transverse momentum [30–35].

In [36] it is pointed out that the low-transverse momentum spectrum for *Z*-boson production at LHC cannot be fully understood without the introduction of TMD, and further study is considered as necessary (see also [37]). The *W*-production is a natural test for the TMD factorization, and an explicit evaluation of the cross section within this approach is yet missing in the literature to our knowledge. On the other side there exist codes and predictions [38–62] for the *W* spectrum that include non-perturbative TMD effects only in a limited way.

The *W*-spectrum is also interesting in order to establish some properties of the TMD like their flavor dependence. In fact, out of all the extractions cited above, only [35] can be sensitive to some non-trivial flavor structure of TMD because it includes also data from semi-inclusive deep inelastic scattering (SIDIS) from Hermes [63] and Compass experiments [64], despite the fact that it analyzes only processes mediated by neutral vector bosons. Some preliminary work in this sense has been performed in [65] and later in [66] where the authors conclude that LHC data on *W*-boson production must be sensitive to the non-trivial flavor structure of TMD. This statement can partially be tested in the present work. We also consider of major importance to well establish the flavor dependence of the TMD before the starting of the Electron Ion Collider (EIC).

The results that we present want to provide a set of predictions for *W*-production which include the latest information from TMD studies. We revise the kinematics of *W*-boson transverse momentum distribution in order to include kinematical power suppressed terms as in [35] and to implement the fiducial cuts typical of these processes. We pay particular attention to errors in our predictions as coming from scale variations, PDF, TMD parameterization whose present knowledge is here described in detail. We recall that the TMD factorization applies to Drell–Yan (DY) type processes for values of the transverse momentum of the vector boson ($q_T$) much lower then its virtual mass (*Q*). The factorization theorem concludes that the non-perturbative parts of the $q_T$-differential cross-sections of boson production when $q_T\ll Q$ are included in the transverse momentum dependent (TMD) distributions and, separately, their evolution kernel [67–77]. Phenomenologically it has been found that the TMD factorization at leading order works for $q_T/ Q\lesssim 0.2$ [32, 34, 35] and we consider this range of validity also here. A theoretical estimate of this range has been provided in [78].

The theoretical perturbative calculations for TMD distributions at small-*b* performed in recent years is highly significative and recently it has reached the $\hbox {N}^3$LO precision^1^ [79–87]. Results of the same $\hbox {N}^3$LO already exist for the universal QCD anomalous dimensions [88–92], so that finally one has an extremely accurate perturbative input. At present the non-perturbative parts of the TMD are extracted at NNLO (which means $\hbox {N}^3$LL in the evolution kernel and NNLO in the matching of the TMD to PDF) so that for consistency we will use the perturbative results up to this order which are included in the code Artemide [93] and the non-perturbative parameters as extracted in [35]. The theoretical settings of this work are very similar to the ones of [35] and we make explicit use of the $\zeta$-prescription [77, 94]. The main difference with respect to the neutral boson case is represented by the particular kinematics of the *W*-production, which we study in detail in the next sections. Thanks to our explicit study of the leptonic tensor and implementation of fiducial cuts we can compare our predictions with existing results from LHC (ATLAS, CMS) and TeVatron (CDF, D). We explore explicitly new regions of the measured phase space where TMD effects can be significative and experimentally testable, especially for di-lepton masses below the *W*-mass peak.

In order to establish some notation we start writing the *W* mediated reaction

1.1 $$\begin{aligned} h_1 h_2 \rightarrow W^+(W^-) \rightarrow l^+(l^-)+\nu _l ({\bar{\nu }}_l), \end{aligned}$$

where $h_{1,2}$ are hadrons (typically protons and antiprotons) and $l^\pm =e^\pm , \mu ^\pm$ and $\nu _l({\bar{\nu }}_l)$ are their corresponding (anti)neutrinos. We consider a cross section differential in the vector boson transverse mass ($m_T$) and transverse momentum ($q_T$). The typical TMD condition $q_T\ll Q$ becomes in this case $q_T\ll m_T$, while the relation among $m_T$ and lepton momenta is highly non-trivial as outlined in the next sections. For our predictions, we consider the case of LHC experiments at $\sqrt{s}=13$ TeV, assuming that lepton cuts and fiducial cross sections are similar to the case of $\sqrt{s}=7$ TeV [25]. In principle this discussion can be extended to other center of mass energies. We consider several intervals of $m_T$ and $q_T$ which are relevant both for the TMD flavor determination and the mass of the *W*-boson. In this sense we discover that several intervals of these variables can be interesting for QCD studies and we investigate them in detail. We consider also the $p_T^Z/p_T^W$ and $p_T^{W^-}/p_T^{W^+}$ observables, in different ranges of the transverse invariant mass of the *W*.

The paper is prepared as in the following. In Sect. 2 we establish the notation and write the cross section of the *W* distributions with TMD factorization and we explain how fiducial cuts are implemented. The kinematical relations are here described in detail (and we are not aware of a similar detailed description in the literature). In Sect. 3 we list the source of errors for the various observables examined in this paper. In Sect. 4 we provide our prediction for *W*, $p_T^{Z}/p_T^W$, $p_T^{W^-}/p_T^{W^+}$ transverse momentum differential distributions. In Sect. 5 we compare the result of our code with existing theoretical and experimental results. We conclude in Sect. 6 and we provide some details of our calculations in the Appendix.

## *W*-boson cross section in TMD factorization

In this section we present the cross section for charged Drell–Yan (DY) process in TMD factorization. Because the derivation of the factorized cross section is analogous to the neutral Drell–Yan case [35, 67–77], we omit a detailed discussion of the factorization, concentrating on the inputs relevant to us.

### Charged DY cross section with transverse variables

Let us consider a charged DY process as in Eq. (1.1) in which two hadrons with momenta $P_1$ and $P_2$ lead to a lepton with momentum *l* and an (anti)neutrino with momentum $l'$. We approximate the interaction among protons and the lepton-neutrino pair through the production of a $W^\pm$ boson with momentum $q=l+l'$. In this paper we do not consider masses of the initial hadrons nor masses of final-state particles, so $P_1^2=M_1^2 \approx 0, \;P_2^2=M_2^2 \approx 0, \; l^2 =m_l^2 \approx 0, \; l'^2=m_{\nu _l}^2 \approx 0.$

The combination of these momenta leads to the definition of the relevant kinematical variables of the DY process

2.1 $$\begin{aligned} s=(P_1+P_2)^2, \qquad q^2=Q^2, \qquad y=\frac{1}{2}\ln \left( \frac{q^0+q^z}{q^0-q^z}\right) ,\nonumber \\ \end{aligned}$$

where *s* represents the square of the center of mass energy, $Q^2$ is the invariant mass of the lepton-neutrino pair and *y* is the rapidity of the produced *W* boson. As the neutrino is a non-detected particle the quantity $Q^2$ is not measurable anymore and one introduces the transverse mass [21, 25, 60, 95–97] as a measurable quantity in processes with invisible particles in final state as

2.2 $$\begin{aligned} m_T^2=2|l_T||l_T'| (1-\cos \phi _{l\nu }), \end{aligned}$$

where $l_T$ and $l_T'$ are the transverse parts of the lepton and neutrino momenta respectively and $\phi _{l\nu }$ is the relative angle between both particles.

The cross section of a general DY process mediated by a gauge-boson *G* can be written as in [35]

2.3 $$\begin{aligned} d\sigma =\frac{\alpha _{\mathrm{{em}}}^2}{2s}d^4 q \sum _{GG'} L_{GG'}^{\mu \nu } W_{\mu \nu }^{GG'} \Delta _G(q) \Delta ^*_{G'}(q), \end{aligned}$$

where $\alpha _{\mathrm{{em}}}=e^2/4\pi$, being *e* the electron charge. This cross section is written in terms of a product of a lepton tensor $L_{GG'}^{\mu \nu }$ and a hadron tensor $W_{\mu \nu }^{GG'}$ defined as

2.4 $$\begin{aligned} L_{\mu \nu }^{GG'}= & {} e^{-2} \left\langle 0 | J_\mu ^G (0) |l,l' \rangle \langle l,l' | J_\nu ^{G'\dagger }(0) |0\right\rangle , \end{aligned}$$

2.5 $$\begin{aligned} W_{\mu \nu }^{GG'}= & {} e^{-2} \int \frac{d^4 x}{(2\pi )^4} e^{-i(x \cdot q)}\nonumber \\&\times \sum _X \langle P_1, P_2 | J_\mu ^{G\dagger } (x) |X \rangle \langle X| J_\nu ^{G'}(0) |P_1,P_2 \rangle , \end{aligned}$$

where $J_\mu ^G$ is the current for the production of a gauge boson G.

On the other hand, $\Delta _G$ is the (Feynman) propagator of the gauge boson, in this case $G=W$ and

2.6 $$\begin{aligned} \Delta _G(q)=\frac{1}{Q^2-M_W^2+i \Gamma _W M_W} \delta _{GW}, \end{aligned}$$

where $M_W$ and $\Gamma _W$ are the mass and width of the *W* boson given in [98], respectively. We do not include electroweak (EW) corrections, which are however calculated in [99].

The *W*-production is usually expressed in terms of the transverse mass. The relation between the invariant and transverse mass in a general case is non-trivial, so we describe it here in detail. We have

2.7 $$\begin{aligned} Q^2=m_T^2+f(l,l'), \end{aligned}$$

and

2.8 $$\begin{aligned} f(l,l')=2 \left[ (l_T^2+l_z^2)^{1/2}(l_T'^2+l_z'^2)^{1/2}-l_zl_z'\right] -2|l_T| |l_T'|\ .\nonumber \\ \end{aligned}$$

In order to include this function in the cross section we introduce the identity

2.9 $$\begin{aligned} 1=\int d m_T^2 \, \delta (Q^2-m_T^2-f(l,l')), \end{aligned}$$

and the cross section in Eq. (2.3) can be rewritten as

2.10 $$\begin{aligned} d\sigma =\frac{\alpha _{\mathrm{{em}}}^2}{2s}d^4q \, dm_T^2 \sum _{GG'} L_{GG'}^{\mu \nu } W_{\mu \nu }^{GG'} \Delta _G(q) \Delta ^*_{G'}(q). \end{aligned}$$

Note that the Dirac delta in Eq. (2.9) is relocated inside the definition of the lepton tensor which simplifies the practical evaluation of the integral.

The next two sections are devoted to the factorization of the hadronic tensor and the final expression for the lepton tensor affected by transverse mass and fiducial cuts. This information will lead us to the final form of the cross section of charged DY within TMD factorization.

### Factorization of the hadronic tensor

In this paper we use the unpolarized part of the hadron tensor from [35] neglecting the masses of both hadrons. Thus, for a generic gauge boson *G* we omit power suppressed higher-twist TMD contributions and we write

2.11 $$\begin{aligned} W_{\mu \nu }^{GG'}= & {} -\frac{g_T^{\mu \nu }}{\pi N_c} |C_V(Q^2,\mu )|^2 \sum _{ff'} z_{ff'}^{GG'}\nonumber \\&\times \int \frac{d^2 {\varvec{b}}}{4\pi } e^{i ({\varvec{q}} \cdot {\varvec{b}})} f_{1,f\leftarrow q}(x_1,{\varvec{b}},\mu ,\zeta )\nonumber \\&\times f_{1,f'\leftarrow q}(x_2,{\varvec{b}},\mu ,\zeta ), \end{aligned}$$

where $C_V$ is the matching coefficient for vector current to collinear/anti-collinear vector and $z_{ff'}^{GG'}$ are the EW factors that will be defined later. On the other hand $f_1$ is the unpolarized TMDPDF defined as

2.12 $$\begin{aligned}&f_{1,f\leftarrow h}(x,{\varvec{b}},\mu ,\zeta )\nonumber \\&\quad =\int \frac{d\lambda }{2\pi } e^{-i x\lambda p^+} \sum _X \langle h(p)|{\bar{q}} (n\lambda + {\varvec{b}} ) W_n^\dagger (n\lambda + {\varvec{b}}) \nonumber \\&\qquad \times \frac{\gamma ^+}{2} |X \rangle \langle X | W_n(0) q(0) |h(p) \rangle , \end{aligned}$$

where *p* is the momentum of the hadron, $W_n(x)$ is a Wilson line rooted at *x* and pointing along vector *n* to infinity. The momentum fraction *x* is defined in DY kinematics as

2.13 $$\begin{aligned} x_{1,2}=\frac{\sqrt{Q^2+q_T^2}}{\sqrt{s}}e^{\pm y}. \end{aligned}$$

In the small-*b* limit, the TMDPDF can be re-factorized in terms of matching coefficients (calculated up to NNLO in [82]) and integrated PDFs. In order to write a complete TMD, we consider also a function modeling non-perturbative effects that are not included into collinear PDFs

2.14 $$\begin{aligned} f_{1,f\leftarrow h}&(x,{\varvec{b}},\mu ,\zeta )= C(x,{\varvec{b}},\mu ,\zeta ) \otimes f_1(x,\mu ) f_{NP}(x,{\varvec{b}}), \end{aligned}$$

where the symbol $\otimes$ represents the Mellin convolution in the *x* variable. We use the ansatz for $f_{NP}$ suggested in [35]

2.15 $$\begin{aligned} f_{NP}(x,{\varvec{b}})=\exp \left( -\frac{\lambda _1(1-x)+\lambda _2 x +x(1-x) \lambda _5}{\sqrt{1+\lambda _3 x^{\lambda _4}{\varvec{b}}^2}}\right) , \end{aligned}$$

where the parameters $\lambda _1, \ldots , \lambda _5$ are extracted from a combined DY+SIDIS fit to data in [35]. In the TMD parameterization of Eq. (2.14) the flavor dependence is in principle contained in the parameters $\lambda _i$ and the PDF. The extraction of [35] has found that, given actual SIDIS data it is sufficient to count flavor dependence in PDF only. While we make predictions using the values of $\lambda _i$ as in [35], it is possible that the *W*-boson measurements that we propose will be able to achieve a better control of these parameters, including a flavor dependence.

The angular part of the Fourier integral in Eq. (2.11) can be solved analytically and we can rewrite the hadron tensor

2.16 $$\begin{aligned} W_{\mu \nu }^{GG'}= & {} -\frac{g_T^{\mu \nu }}{\pi N_c} |C_V(Q^2,\mu )|^2 \sum _{ff'} z_{ff'}^{GG'} W^{ff'}_{f_1 f_1}\nonumber \\&\times (Q, q_T,x_1,x_2, \mu , \zeta ), \end{aligned}$$

where

2.17 $$\begin{aligned}&W^{ff'}_{f_1 f_1}(Q, q_T,x_1,x_2, \mu , \zeta )\nonumber \\&\quad =\int \frac{|{\varvec{b}}|d |{\varvec{b}}|}{2} J_0(|{\varvec{b}}| |{\varvec{q}}|) f_{1,f\leftarrow q}(x_1,{\varvec{b}},\mu ,\zeta )\nonumber \\&\quad \times f_{1,f'\leftarrow q}(x_2,{\varvec{b}},\mu ,\zeta ). \end{aligned}$$

### Lepton tensor and fiducial cuts

The lepton tensor that enters in the cross section is integrated over the lepton and neutrino momenta, thus contracting it with the Lorentz structure that comes from the hadron tensor in Eq. (2.11) we obtain,

2.18 $$\begin{aligned}&-g_T^{\mu \nu }L_{\mu \nu }^{GG'(\mathrm{cuts})}\nonumber \\&\quad =32 z_{ll'}^{GG'}\int \frac{d^3l}{2E}\frac{d^3l'}{2E'} [ll'-(ll')_T]\theta (\mathrm{cuts}) \delta ^{(4)}(l+l'-q)\nonumber \\&\qquad \times \delta (Q^2-m_T^2-f(l,l')), \end{aligned}$$

where the extra label *cuts* represents the fiducial cuts introduced in different experiments. These cuts are implemented over the momenta of lepton and neutrino and the rapidity of the lepton

2.19 $$\begin{aligned} \eta _{\mathrm{{min}}}< \eta <\eta _{\mathrm{{max}}}, \qquad l_T^2> p_1^2, \qquad l_T'^2 >p_2^2. \end{aligned}$$

On the other hand note that the difference of this lepton tensor with the one for neutral DY production [32, 35] is the extra dependence on the transverse mass through the delta function introduced in Eq. (2.9). The integral in Eq. (2.18)

2.20 $$\begin{aligned}&I_W(Q^2,m_T^2,q_T)\nonumber \\&\quad =\int \frac{d^3l}{2E}\frac{d^3l'}{2E'} [ll'-(ll')_T] \theta (\mathrm{cuts}) \delta ^{(4)}(l+l'-q)\nonumber \\&\qquad \times \delta (Q^2-m_T^2-f(l,l')), \end{aligned}$$

cannot be solved analytically and only numerical results can be obtained. A detailed discussion about this integral can be found in Appendix A.

### Final expression of the cross section

Once we have precise definitions for hadron and lepton tensor and taking into account that

2.21 $$\begin{aligned} d^4 q= \frac{\pi }{2} dQ^2 dy d q_T^2, \end{aligned}$$

we get the desired differential cross section as

2.22 $$\begin{aligned} \frac{d\sigma }{dm_T^2 dy d q_T^2}&=\int _0^\infty dQ^2 \frac{8}{N_c} \frac{\alpha ^2_{\mathrm{{em}}}}{s} I_W(Q^2,q_T, m_T^2)\nonumber \\&\quad \times \sum _{ff'}\sum _{GG'}z_{ll'}^{GG'} z_{ff'}^{GG'} \Delta _G (q) \Delta _{G'}^* (q) W_{f_1f_1}^{ff'} \nonumber \\&\quad \times (Q^2, q_T,x_1,x_2). \end{aligned}$$

where, in the case of *W* boson production, the product of EW factors for hadronic and leptonic part and propagators can be written as

2.23 $$\begin{aligned}&\!\!\!z_{ll'}^{GG'} z_{ff'}^{GG'}\Delta _G(q)\Delta _{G'}^*(q)\nonumber \\&\!\!\!\quad =\delta _{GW}\delta _{G'W} |V_{ff'}|^2 \frac{e_f e_{f'}}{Q^4} \frac{Q^4}{(Q^2-M_W^2)^2+\Gamma _W^2 M_W^2} z_l^{WW} z_q^{WW},\nonumber \\ \end{aligned}$$

where $V_{ff'}$ are the elements of CKM matrix that mixes flavors, $e_f$ are the quark charges in terms of the electron charge and

2.24 $$\begin{aligned} z_l^{WW} z_q^{WW}=\left( \frac{1}{4s_W^2}\right) ^2. \end{aligned}$$

Thus, the final expression for the desired cross section is

2.25 $$\begin{aligned} \frac{d\sigma }{dm_T^2 dy d q_T^2}= & {} \int _0^\infty \frac{dQ^2}{Q^4}\frac{8}{N_c} \frac{\alpha ^2_{\mathrm{{em}}}}{s} I_W(Q^2,q_T,m_T^2) \nonumber \\&\times \frac{1}{(4s_W^2)^2} \frac{Q^4}{(Q^2-M_W^2)^2+\Gamma _W^2 M_W^2}\nonumber \\&\times \sum _{ff'} |V_{ff'}|^2 e_f e_{f'} W_{f_1f_1}^{ff'} (Q^2, q_T,x_1,x_2).\nonumber \\ \end{aligned}$$

The plots of next sections are done using the PDF set of NNPDF31_nnlo_as_0118 [28]. The numerical inputs that we have used are

$$\begin{aligned} M_Z&=91.1876\text { GeV},\quad M_W=80.379\text { GeV},\quad \\ \Gamma _Z&=2.4952\text { GeV},\quad \Gamma _W=2.085\text { GeV},\quad \end{aligned}$$

and $$G_F=1.1663787\times 10^{-5}$$ GeV.

## Sources of errors

In the course of the paper we consider several sources of errors that come from the way we write and parameterize the cross section. We list them here as a reference for the following sections.

- **Scale variations** In the present work we use the $\zeta$-prescription defined in [77] and we include the non-perturbative fixing of the $\zeta$-scale as defined in [35, 94]. As a result the scale variation is done changing the parameters $c_{2,4}\in [0.5,2]$. These parameters define the uncertainty in the hard matching scale and in the matching of the TMD on the collinear PDF respectively.
- **Error from a reference PDF set using replicas** The central value of our predictions is deduced using the PDF set NNPDF31_nnlo_as_0118 [28], which includes also LHC data. The error on each bin is evaluated taking the variance over 1000 replicas.
- **Predictions from different PDF sets** In [35] the authors have performed fits of DY data at low and high energy with different sets of PDF and we report their results in Appendix B. We consider here the same sets of PDF and we show how the predictions change with these different sets.
- **Error from TMD parametrization** In [35] the authors provide a set of replicas of their TMD parameterization and we provide the main estimation of this error using them. Alternatively, one could use a more standard error propagation coming from the estimate of the non-perturbative parameters. We have used this second method as a check, and we provide more details in Appendix C.

## Observables in *W* production with TMD

In this section we concentrate on some observables that can be, in principle, sensitive to TMD effects and/or are relevant to establish some important properties of the *W* as its mass. We consider the *W*-boson differential cross section, the ratios for $p_T^Z/p_T^W$, and $p_T^{W^-}/p_T^{W^+}$ all of them as a function of the boson transverse momentum distribution and different intervals of the transverse mass. The fiducial cuts have been set as the ATLAS experiment [25] but for $\sqrt{s}=13$ TeV. We have identified three interesting intervals of $m_T$, namely [50,66] GeV, [66,99] GeV, [99,120] GeV corresponding to $m_T$ values below, around, above the *W*-mass, with typical cuts on lepton momenta. The $m_T$-interval which is mostly studied in the literature is the one with $m_T\in [66,99]$ GeV, however this is not the only interesting one from the perspective of studying the TMD properties. This is because non-perturbative TMD effect are expected to be more relevant for low values of $m_T$, similarly to Drell–Yan processes. The $q_T$ interval that we have considered, that is $q_T/m_T\lesssim 0.2$ is consistent with the application for TMD factorization. In order to have a reference setup for PDF we have chosen NNPDF31_nnlo_as_0118 [28] as in [35] as it is one of the sets that include LHC data. Nevertheless we have also examined the results taking into account different sets. We have considered a $q_T$ binning of 1 GeV, which much probably exceeds the possibilities of current experiments. We motivate this choice because it has been used also by other authors, see f.i. [60], and also because it is allows to better evidence the TMD effects.

### Spectra of the $W^\pm$

**Fig. 1:**
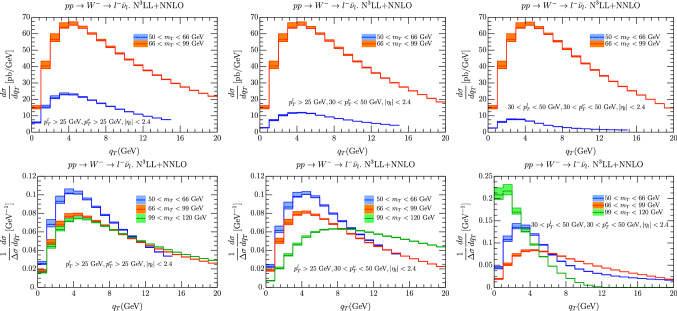
(top) Unnormalized cross sections for $W^-$ production with different intervals of lepton cuts. (bottom) Normalized cross sections for $W^-$ production with different intervals of lepton cuts

We have considered the spectra of the $W^\pm$ and in Fig. 1 we show the case for the $W^-$ (similar plots are obtained for $W^+$). In the upper panels of Fig. 1 we have considered three possible cuts for lepton momenta as reported in the same figures. In the upper panels of Fig. 1 the cross section for $m_T\in [99,120]$ GeV is not reported because it is much smaller than the others. In the same figure we have included error bands corresponding to scale variations. The figures in these panels show that the cuts have marginal impact for $m_T\in [66,99]$ GeV while they can suppress the cross section in the lower interval $m_T\in [50,66]$ GeV. The difference in the values of the cross sections however is about a factor 4. The interval $m_T\in [99,120]$ GeV instead results to be even more suppressed an it is not shown in the figure. Given this suppression and the fact at high values of $m_T$ all TMD effects are washed out we neglect it in the rest of the figures.

In the lower panels of Fig. 1 we normalize the cross section to its value integrated in $q_T$ in the interval shown in the figure. The shape of the curves now changes and in the left and central panels the more peaked cross sections is the one obtained for $m_T\in [50,66]$ GeV, which is partly due to the fact that we always select $q_T/m_T\le 0.2$.

In order to drive a conclusion from these plots we have to recall the previous experience of the fit of [34, 35] and also the results of [36]. In these works it shown that in TMD analyses increasing the value of *Q* the non-perturbative QCD effects are washed out. As a a result having data below the $W,\, Z$ boson peaks is extremely useful for this kind of research and it can provide valuable information. The plots shown in Fig. 1 actually show that within the current facility it is possible to achieve this goal. The bands shown in Fig. 1 come from scale variations.

**Fig. 2:**
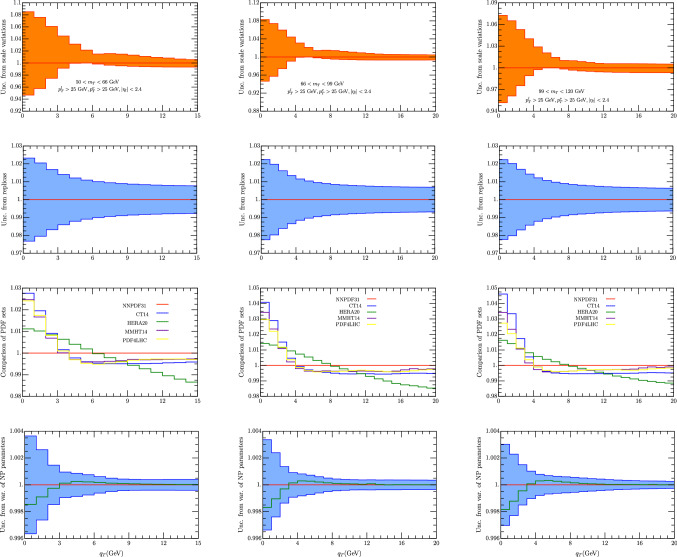
Error for $W^-$ cross section for $p_T^{l,\nu }>25$ GeV, $|\eta _l|<2.4$ and the $m_T$ intervals [50, 66] GeV (left column), [66, 99] GeV (middle column), [99, 120] GeV (right column). In the first line we report the theoretical error from scale uncertainties as explained in the text. In the second line we have the error calculated as a variance in each bin of 100 replicas of the set NNPDF31_nnlo_as_0118 [28]. The uncertainty is referred to the average value of each bin (red line). The value of the cross section given by the central replica is represented by the green line. On the third line we represent the value of each been with different sets of PDF. On the fourth row we have the uncertainty due to non-perturbative parameters. The central value is given by the the central replica of NNPDF31_nnlo_as_0118 [28]

**Fig. 3:**
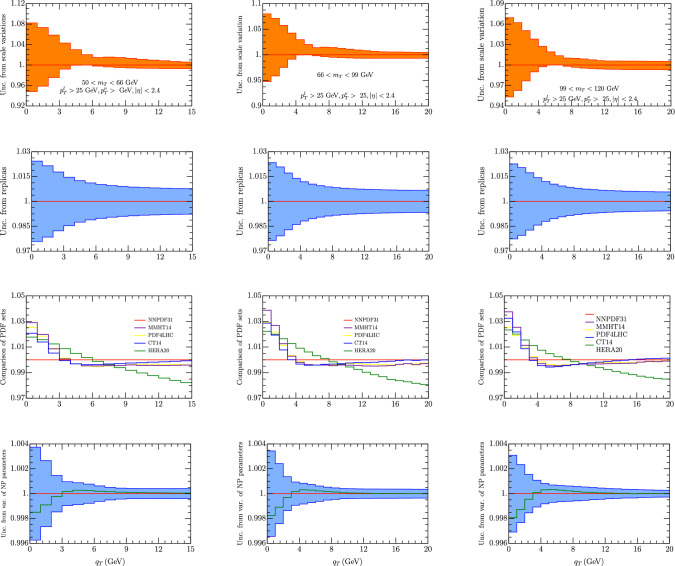
Error for $W^+$ cross section for $p_T^{l,\nu }>25$ GeV, $|\eta _l|<2.4$ and the $m_T$ intervals [50, 66] GeV (left column), [66, 99] GeV (middle column), [99, 120] GeV (right column). In the first line we report the theoretical error from scale uncertainties as explained in the text. In the second line we have the error calculated as a variance in each bin of 100 replicas of the set NNPDF31_nnlo_as_0118 [28]. The uncertainty is referred to the average value of each bin (red line). The value of the cross section given by the central replica is represented by the green line. On the third line we represent the value of each been with different sets of PDF. On the fourth row we have the uncertainty due to non-perturbative parameters. The central value is given by the the central replica of NNPDF31_nnlo_as_0118 [28]. The cross section for the central replica is shown in Fig. 7

In Figs. 2 and 3 we have studied the errors as listed in Sect. 3 for one particular set of fiducial cuts for $W^-$ and $W^+$ respectively. Similar errors are obtained also in the other cases. Going from the top row to the bottom one in Figs. 2 and 3 one finds: (1) the theoretical error from scale uncertainties; (2) the error calculated as a variance in each bin of 1000 replicas of the set NNPDF31_nnlo_as_0118 [28]. Here the bands are referred to the average value of each bin (red line). The value of the cross section given by the central replica is represented by the green line; (3) the uncertainty as coming from different sets of PDF; (4) the uncertainty due to non-perturbative parameters: in this case the central line is given by the the central replica of NNPDF31_nnlo_as_0118 [28].

There is a series of observations that one can make on these plots. As a first the smallest variations are provided by replicas and non-perturbative parameters and both of them are below $1\%$. The scale variation is certainly the biggest source of error: it is in range 2–9$\%$ for $q_T\le$ 4 GeV, and about 1–2$\%$ for $q_T\ge$ 4 GeV. We recall that the present analysis uses $\hbox {N}^3$LL for TMD evolution and NNLO for all the rest. In the introduction it was pointed out that recently higher order perturbative calculations have been performed. It will be interesting to observe how the scale variation changes when including this higher order term. On the other side before doing this one should have a TMD extraction of the same order.

In exploring different PDF extractions we have considered the ones for which a TMD extraction has been provided in [35] . We recall that the HERA PDF [100] actually provided the best fit in that case. Observing Figs. 2 and 3 one sees that actually this set behaves differently with respect to the others, however the difference with NNPDF31 is below 1-2 $\%$ an all over the range of *qT* that we have considered. For all the other sets, there is a major difference with NNPDF31 for $q_T\le$4 GeV while they agree within 1$\%$ for greater values of $q_T$.

The uncertainties due to non-perturbative parameters result to be almost negligible. The non-perturbative parameters have uncertainties coming from their extraction from data, that is from a $\chi ^2$ analysis of fits. Despite the fact that some of these uncertainties may be significative on each single parameter the impact of each single variation is just a fraction of the impact of the whole non-perturbative TMD structure because their value is significantly different from zero. It is also possible that the errors on each parameter is under-estimated as suggested in [35].

### Ratio $p_T^Z/p_T^W$

**Fig. 4:**
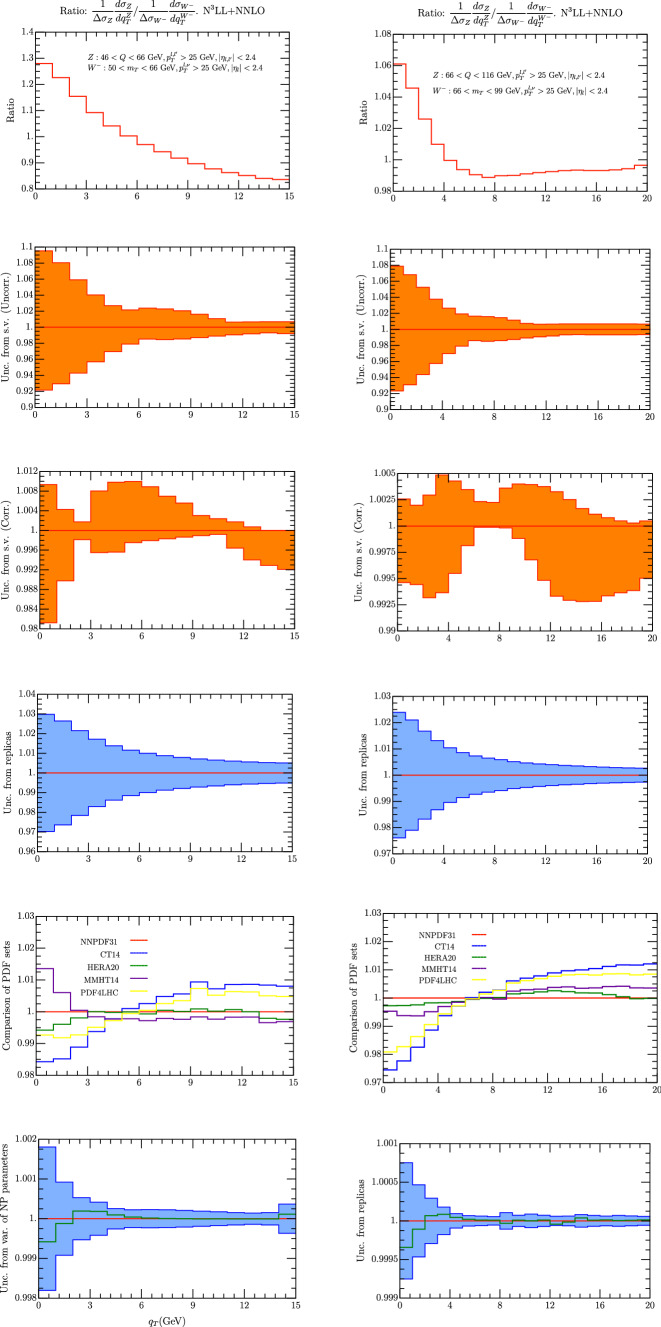
Ratio of $Z/W^-$ spectrum for $m_T\in [50,66]$ GeV (left column) , and $m_T\in [66,99]$ GeV (right column). On the first row we have the $Z/W^-$ spectrum using the central replica of NNPDF31_nnlo_as_0118 [28]. Uncorrelated and correlated theoretical uncertainties are given in second and third row respectively. In the third line we have the error calculated as a variance in each bin of 100 replicas of the set NNPDF31_nnlo_as_0118 [28]. The uncertainty is referred to the average value of each bin (red line). The value of the observable given by the central replica is represented by the green line. On the fourth line we represent the value of each been with different sets of PDF. On the fifth row we have the uncertainty due to non-perturbative parameters

**Fig. 5:**
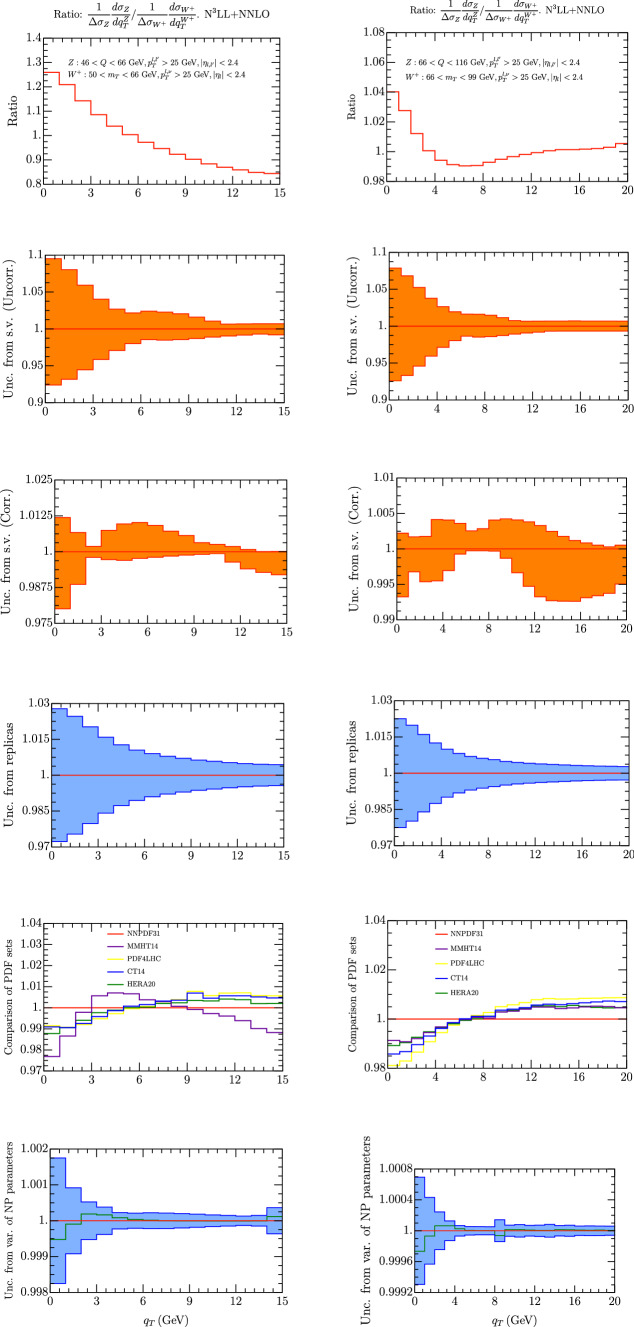
Ratio of $Z/W^+$ spectrum for $m_T\in [50,66]$ GeV (left column) , and $m_T\in [66,99]$ GeV (right column). On the first row we have the $Z/W^-$ spectrum using the central replica of NNPDF31_nnlo_as_0118 [28]. Uncorrelated and correlated theoretical uncertainties are given in second and third row respectively. In the third line we have the error calculated as a variance in each bin of 100 replicas of the set NNPDF31_nnlo_as_0118 [28]. The uncertainty is referred to the average value of each bin (red line). The value of the observable given by the central replica is represented by the green line. On the fourth line we represent the value of each been with different sets of PDF. On the fifth row we have the uncertainty due to non-perturbative parameters

It has been pointed out since a long time that the observable

4.1 $$\begin{aligned} \left( \frac{1}{\Delta \sigma ^{Z}} \frac{d\sigma ^{Z}}{\, dq_T} \right) /\left( \frac{1}{\Delta \sigma ^W}\frac{d\sigma ^W}{\, dq_T} \right) \text { vs. } q_T \end{aligned}$$

can show a very small uncertainty because the PDF contributions tend to cancel and it can be useful for the measurements of the *W* mass (some recent phenomenological work can be found in [101, 102]). The region of $q_T$ where this observable is actually measured corresponds to the region of validity of the TMD factorization theorem, so it is interesting to observe what is the impact of TMD. In formula 4.1 the numerator and the denominator are weighted by $\Delta \sigma ^{W,Z}$ that is the cross section integrated in the $q_T$ interval under study. This is preferable to normalizing to the total cross section $\sigma ^{W,Z}$ which should be extracted elsewhere. The result of our prediction is shown in Figs. 4 and 5 for $W^-$ and $W^+$ respectively. We have considered two intervals of $m_T$, being the lower one useful to control better TMD effects and several sources of error as in the previous section. The scale uncertainty now is treated as in [60], considering separately the correlated and uncorrelated cases.

The error from scale variations can be considered as correlated or uncorrelated as in [60], see second and third row in Fig. 4. The uncorrelated uncertainty is well below 1$\%$ for the central interval and most of the low interval of $m_T$. On the other side the correlated uncertainty is below 2$\%$ only for $q_T\ge$ 4 GeV. A similar trend is shown when different sets of PDF are used. In this case the spread of the results can be at most 2$\%$ for $q_T\le$ 4 GeV, and at most 1$\%$ for $q_T\ge$ 4 GeV. We find remarkable that the PDF error results slightly inferior for the low $m_T$ interval, which confirms that this is an interesting case to study. Another interesting observation is that the difference between the HERA and NNPDF31 sets is always well below 1$\%$. The other sources of error that we have considered, give uncertainties less the 1$\%$ on all over the intervals that we have studied.

### Ratio $p_T^{W^-}/p_T^{W^+}$

**Fig. 6:**
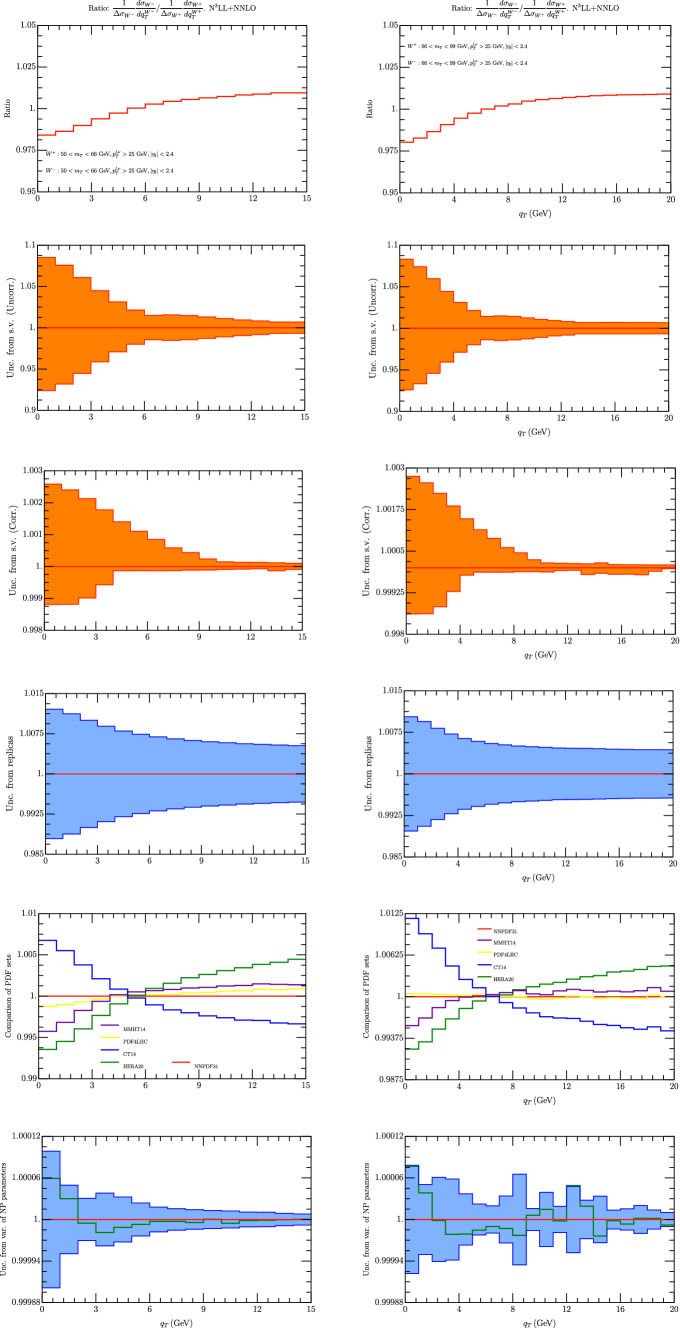
Ratio of $W^-/W^+$ spectrum for $m_T\in [50,66]$ GeV (left column) , and $m_T\in [66,99]$ GeV (right column). On the first row we have the $W^-/W^+$ spectrum using the central replica of NNPDF31_nnlo_as_0118 [28]. Uncorrelated and correlated theoretical uncertainties are given in second and third row respectively. In the third line we have the error calculated as a variance in each bin of 100 replicas of the set NNPDF31_nnlo_as_0118 [28]. The uncertainty is referred to the average value of each bin (red line). The value of the observable given by the central replica is undistinguishable from the red line. On the fourth line we represent the value of each been with different sets of PDF. On the fifth row we have the uncertainty due to non-perturbative parameters

The ratio of $W\pm$ cross sections shown in Fig. 6. We have considered again two intervals of $m_T$ and it is nice to observe the similarity of these curves in the two intervals. The theoretical errors have been estimated and scale variations represent the biggest error. We have considered both correlated and uncorrelated scale variations and their value is very similar in this case. The scale variations keeps below 2$\%$ for $q_T>4$ GeV and grows below this value. All other uncertainties keep below the 1$\%$.

## Comparisons with other groups and experiments

**Fig. 7:**
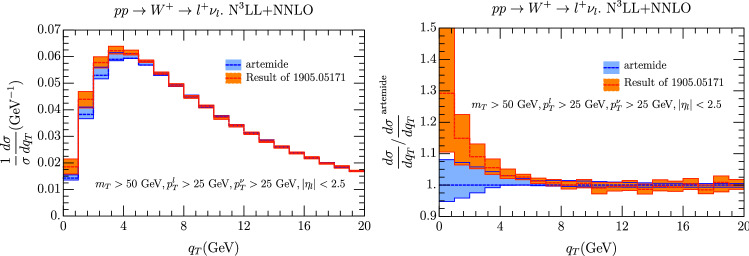
Comparison of our prediction with [60] including errors coming from scales variation. On the right the two cross sections are normalized to the central value of the prediction from Artemide

**Fig. 8:**
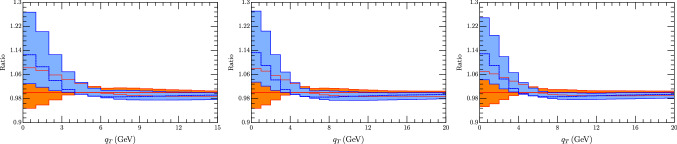
Ratio of $W^+$ cross section without $\lambda _i$ non-perturbative effects over the same cross section with the full model (blue band). The scale errors are shown by the bands. The orange band is ratio of the cross section in the full model over itself, and scale error band is also shown. The left, central, right panels correspond respectively to the cases of $50\; \mathrm{GeV}< m_T<66; \mathrm{GeV}$, $66\; \mathrm{GeV}< m_T<99; \mathrm{GeV}$, $99\; \mathrm{GeV}< m_T<120; \mathrm{GeV}$

The study of TMD in *W*-production has not been explored in its full potential in the literature, because the formulation of the TMD factorization theorem with full details is very recent. The main difficulty in establishing this kind of studies is that the TMD factorization only holds for $q_T\ll Q$, and in this *W*-case $q_T\ll m_T$. In this way we are interested in describing only a (relevant) part of the spectrum, the one which contains the peak of the distribution.

In this section we would like to compare the outcome of the results as coming from TMD factorization and recent TMD fits with theoretical and experimental results.

A theoretical prediction of the LHC case can be found in [60] and the comparison with us is done in Fig. 7. In this plot we show the cross section for the two groups in the interval 0 GeV $<q_T <20$ GeV. The cross section is normalized to the total cross section as provided by [60], in order to have a reference value. The scale uncertainty in the two curves is similar, although slightly reduced in our case. There can be multiple reasons for this, like a different choice of scales or the usage of Monte Carlo calculation in [60]. One observes also a remarkable difference in the central values of the cross section for very low values of $q_T\le 5$ GeV. The origin of the difference may have multiple motives starting from the parametrization of the non-perturbative effects (which is based on a broader data analysis in our case) to the set of prescription and scale fixing used by the two groups. In general this difference is expected where non-perturbative effects are significative as it is shown by the figure, while it is much less significative for $q_T\ge 5$ GeV. In order to explore this we have considered a modified version of the TMD where the effect of the constants $\lambda _i$ in Eq. (2.15) is nullified. This is achieved considering $f_{NP}(x,{\varvec{b}})=\exp (-\lambda b^2)$ with $\lambda \simeq 10^{-3}$. The results are shown in Fig. 8 and they show to be consistent with Fig. 7 where the TMD non-perturbative part was not considered, despite the fact that the kinematical cuts considered in the two figure are slightly different. We postpone anyhow a deeper study to a different work.

**Fig. 9:**
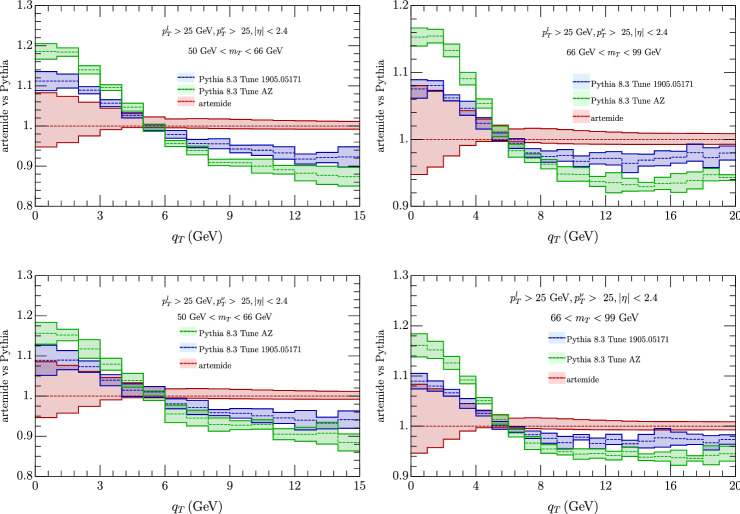
Comparison of Artemide cross section with Pythia 8.3 AZ tune as in [60] (blue band) and as in the original ATLAS release (green band) for $W^+$ (top panels) and $W^-$ (low panels). The Artemide error comes from scale variations, the Pythia errors are commented in the text

**Fig. 10:**
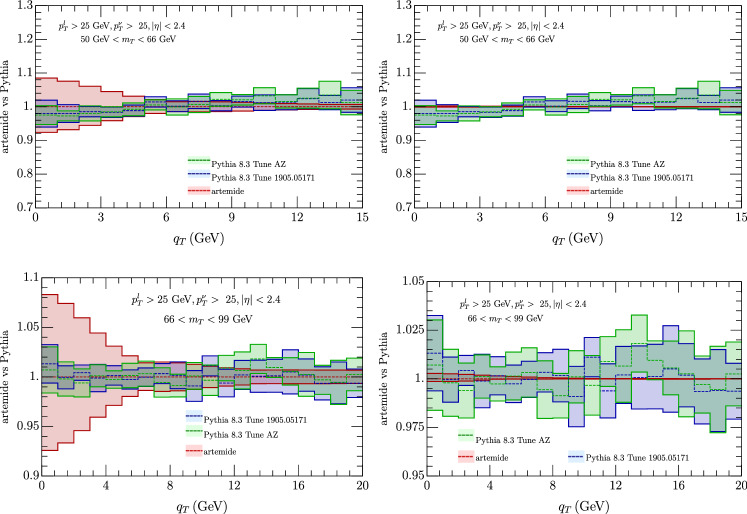
Comparison of Artemide $p_T^{W^+}/p_T^{W^-}$ ratio with Pythia 8.3 AZ tune as in [60] (blue band) and as in the original ATLAS release (green band). Left panels show uncorrelated errors and right panels the correlated ones. The Artemide error comes from scale variations, the Pythia errors are commented in the text

**Fig. 11:**
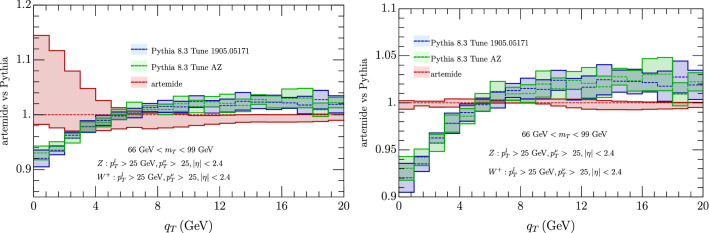
Comparison of Artemide $p_T^Z/p_T^{W}$ ratio with Pythia 8.3 AZ tune as in [60](blue band) and as in the original ATLAS release (green band).. Left panels show uncorrelated errors and right panels the correlated ones. The Artemide error comes from scale variations, the Pythia errors are commented in the text

We have considered also a comparison with Pythia 8.3 [103], with AZ tune as adapted by ATLAS experiment. We have found two version of this tune one as used in [60]^2^ In the comparison of the cross sections Fig. 9 we observe a general shift of the Pythia result with respect to ours, whose sign depends on the value of $q_T$, although the two estimates are mostly compatible within the errors (more for 66 GeV $<m_T<$ 99 GeV than in the less energetic interval). In the ratio of $W^\pm$ cross section (Fig. 10) instead the agreement is complete, both considering correlate and uncorrelated errors, providing Artemide a smaller error band. Similar conclusion come from the $p_T^Z/p_T^W$ in Fig. 11, although with more difference for transverse momentum less than 5 GeV.

**Fig. 12:**
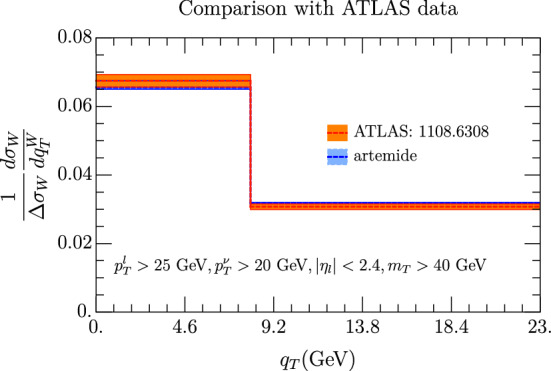
Comparison of our prediction with data from ATLAS in [95, table II], including errors coming from scales variation

Current experiments at LHC at the moment have a limited number of data points. For ATLAS we have found that for the low $q_T$ region, one has only two points in [95], shown in Fig. 12. The cross section is normalized to its integrated value in the plotted $q_T$ interval. The theoretical and experimental values are compatible within the errors. However it is evident that such a large binning does not allow a precise statement.

**Fig. 13:**
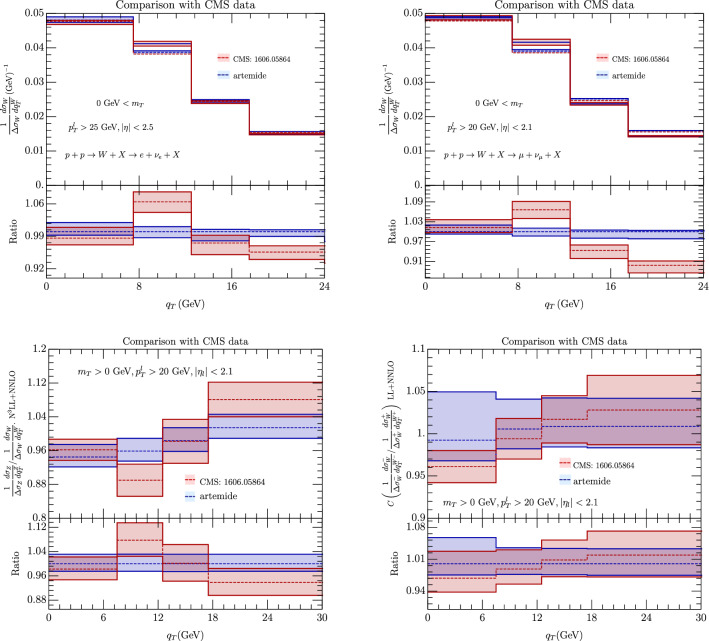
Comparison of our prediction with data from CMS in [21]. Theoretical predictions include error coming from scales variation. The CMS data are always evaluated for $p_T^l> 25$ GeV, $p_T^\nu > 20$ GeV, $|\eta _l|<2.4$.(Top left panel) *W* boson normalized spectrum for electron final state, (top right panel) *W* boson normalized spectrum for muon final state, (bottom left panel) *W*/*Z* ratio of transverse momentum normalized spectrum, (bottom right panel) $W^+/W^-$ ratio of transverse momentum normalized spectrum

The CMS experiment has published an analysis of data for the $s=\sqrt{8}$ TeV run in [21]. The data published by this collaboration are totally inclusive in $m_T$, however because of the cuts on leptons, the values of $m_T\le 20$ GeV give small contributions. In Fig. 13 we consider the cases of the *W*, *Z*/*W* and $W^-/W^+$ transverse momentum spectrum comparing our results with [21]. The large bin for $q_T>18$ GeV is the most sensitive to power corrections which however do not seem to create particular problems. The first thing that one can observe is that there is a good agreement between data and our prediction when theoretical errors are included, which suggests the fact that most of the QCD corrections in the CMS experiment are due to the TMD region.

**Fig. 14:**
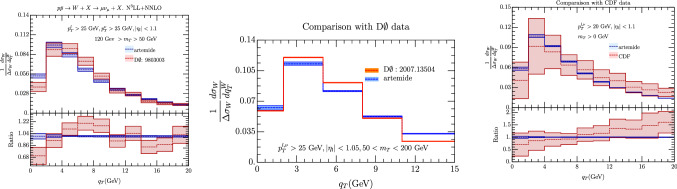
Comparison of our prediction with data from  experiment at $\sqrt{s}=1.8$ TeV [7] and$\sqrt{s}=1.96$ TeV [12] and with CDF experiment at $\sqrt{s}=1.8$ TeV [13]. Theoretical predictions include errors coming from scales variation

Going to TeVatron experiments we have found data for  and CDF and we show a comparison with our prediction in Fig. 14. In the central plot of this figure we do not show the experimental error, because the uncertainty of the bins are correlated through non-trivial correlation matrices. The agreement with these experiments is in general greater than with LHC, mainly due to the larger experimental error as the following considerations remark.

We have considered the cross sections $\chi ^2$ for the different experiments with different sets of PDF and the results are shown in Table 1 (we also report the number of relevant points for each experiment). The CDF result are the ones with larger errors and TMD predictions agrees with them while for the  at $\sqrt{s}=1.8$ GeV, the agreement is worse. We have not considered the case of  at $\sqrt{s}=1.96$ GeV, because the computation of the error in this case involves the knowledge of the W-spectrum up to $q_T=600$ GeV, which we do not have. In the case of LHC, in general we have a very limited number of points for each experiment. ATLAS has a remarkable agreement with the HERA20 PDF sets, definitely better than with other sets. The CMS case is more elaborate. For the case of electronic decay of W at CMS, the high value of $\chi ^2$ is basically driven by just one point out of four, as can be seen Fig. 13, left panel (it is the point in the bin 7.5 GeV$<q_T<$ 12.5 GeV). Removing this point, the $\chi ^2$ is very similar to the ATLAS case. For the muon channel instead we cannot find a particular point which is responsible for the high $\chi ^2$. As a final remark we recall that the CMS observable is totally inclusive on $m_T$, which does not allow a perfect control of factorization hypothesis at the theoretical level. We have checked the impact on the $\chi ^2$ of the theoretical errors (scale uncertainty and PDF replicas) for the case of NNPDF and HERA20 and we have reported it in Table 2. In both cases the errors on each bin are considered uncorrelated to the rest. The scale uncertainty error is dominated by the variation of $c_4$, the parameter which associated to the scale at which TMD are matched onto PDF. We observe that including this error we have a big reduction of the $\chi ^2$ on all experiments. In order to understand better this issue it would help to have experimental results with a definite interval of $m_T$ and also a TMD extraction that includes the *W* processes, that we postpone to a future work. Concerning this last point we have compared the $\chi ^2$ as coming from different extractions of TMD with the code *artemide*. The different extractions are obtained with the NNPDF31, PDF set, and using different data sets as shown in Table 3. We find a substantial agreement among all extractions, which suggests a mild flavor dependence of the TMD. In Table 2, the muon channel in CMS is still not fully agreeing with the theoretical prediction. This fact, which is put in evidence here for the first time, needs further study beyond the present work.

**Table 1:** $\chi ^2/N$ using the extraction of TMD of [35]. For the CMS case $W\rightarrow e \nu$ in parenthesis we also report the $\chi ^2$ with one less point as explained in text

	CDF $\sqrt{s}=1.8$ TeV	D0 $\sqrt{s}=1.8$ TeV	ATLAS	CMS $e\nu$	CMS $\mu \nu$
Number of points	10	10	2	4(3)	4
NNPDF31	0.650	1.845	1.565	7.284 (1.694)	21.502
HERA20	0.617	2.009	0.853	6.024 (0.310)	16.090
MMHT14	0.667	2.166	1.406	7.465 (1.505)	21.751
CT14	0.677	2.608	1.324	7.974 (1.482)	21.972
PDF4LHC	0.660	2.061	1.405	7.733 (1.605)	22.075

**Table 2:** $\chi ^2/N$ using the extraction of TMD of [35] and theoretical errors (which include scale variation and PDF error coming from 1000 replicas)

	CDF $\sqrt{s}=1.8$ TeV	D0 $\sqrt{s}=1.8$ TeV	ATLAS	CMS $e\nu$	CMS $\mu \nu$
Number of points	10	10	2	4	4
NNPDF31	0.540	1.485	0.463	1.674	3.165
HERA20	0.469	1.591	0.271	1.563	3.721

**Table 3:** $\chi ^2/N$ using extractions from different data sets. The PDF is NNPDF31. The first line is the same as in Table 4. For all extractions we have used the NNPDF31 PDF set

Ref. of fit and data set	CDF $\sqrt{s}=1.8$ TeV	D0 $\sqrt{s}=1.8$ TeV	ATLAS	CMS $e\nu$	CMS $\mu \nu$
[35] SIDIS+DY	0.650	1.845	1.565	7.284	21.502
[33] DY	0.651	2.003	1.549	7.783	22.302
[33] DY (high energy)	0.627	1.326	1.999	6.347	20.923
Case 4 of [36] (LHC)	0.694	2.312	1.333	7.681	21.704

## Summary and outlook

In this work we have set the status of the current knowledge of the *W*-boson spectrum within the TMD factorization formulation of its cross section and using the latest extraction of these distributions in [35]. We have considered the *W*-production differential cross section, the $p_T^Z/p_T^W$ and the $p_T^{W^+}/p_T^{W^-}$ distributions as functions of the boson transverse momentum $q_T$ in different intervals of $m_T$. The kinematic of the processes has been deeply studied, providing details that were not treated in the literature (to our knowledge), and showing explicitly how fiducial cuts are implemented together with kinematical power corrections. The kinematical description so achieved has put us in the condition to use the TMD extraction made in [35] with the same level of precision. The perturbative inputs are $\hbox {N}^3$LL for the evolution and NNLO for all the rest. Because of our experience with *Z*-boson production we have given particular attention to values of $m_T$ below ([50,66] GeV) and around the *W*-mass ([66,99] GeV). In fact one expects to better control the non-perturbative QCD effects once the details of the observables below the *W*-mass are also known. It is remarkable that observables at low values for $m_T$ have been poorly studied despite the fact that there is no particular suppression of the cross section and all errors look very similar.

An important chapter is the error analysis. We have considered as sources of error, scale variations, replicas uncertainty within the NNPDF31 PDF set, uncertainties due to different sets of PDF and the ones due to TMD non-perturbative parameterization. This analysis is interesting because it gives a first step in our understanding the errors. We consider it an important starting point that can certainly be improved with future work within TMD studies. For instance scale uncertainties can be checked including one more perturbative order (including very recent results at $\hbox {N}^3$LO, [83–87]), which however require a new TMD extraction. Then, the sets of PDF that we have checked are the ones analyzed also in [35], which is still limited. More PDF sets should be used and included in the TMD extraction. The actual impact of each source of error depends on the observable, and we have described it in Sect. 4.

At present the available data provide already some information on the observables that we have studied. We have considered the cases of TeVatron and LHC. The agreement in the cross section is reasonable for CDF, D at $\sqrt{s}=1.8$ TeV, ATLAS, while it is not completely satisfactory CMS. For all these cross sections we have done a $\chi ^2$ analysis. The message is that inferring and estimating QCD non-perturbative contributions to *W* processes from neutral boson mediated ones has some effectiveness and that a more global treatment of data is also worth studying. This raises also the question of whether a more sophisticated TMD flavor dependence can improve the agreement with data. Other effects like QED contributions [104, 105] will also be addressed in future studies.

In order to perform this study we have proposed to consider several intervals of $m_T$, for which we do not observe a particular suppression of the cross section. Comparing experiments, we find a striking difference between LHC and TeVatron data in the $q_T$ binning of the final result. Definitely the 2 GeV binning of TeVatron allows a much better understanding of the QCD effects, despite the LHC precision. A similar binning would be also desirable for LHC. This problem has already been discussed also in the case of Drell–Yan in [36].

## Data Availability

This manuscript has associated data in a data repository. [Authors’ comment: The original source code from which data can be derived will be published on the official Artemide web site: https://github.com/VladimirovAlexey/artemide-public.]

## Explicit expression for the lepton tensor integration

The integral involved in the definition of the lepton tensor in Eq. (2.18) is

A.1 $$\begin{aligned} I_W(Q^2,m_T^2,q_T)&=\int \frac{d^3l}{2E}\frac{d^3l'}{2E'} \, \delta ^{(4)}(l+l'-q)\, \delta (Q^2-m_T^2\nonumber \\&\quad -f(l,l'))\theta (\text {cuts})\left[ ll'-(ll')_T\right] . \end{aligned}$$

Integrating the momentum of the neutrino with the help of the conservation momentum delta function we can rewrite Eq. (A.1) as

A.2 $$\begin{aligned} P_W(Q^2,m_T^2,q_T)&=\int \frac{d^3l}{2E} \delta ((q-l)^2)\, \delta (Q^2-m_T^2\nonumber \\&\quad -f(l,q))\theta (\text {cuts})\nonumber \\&\quad \times \left[ l(q-l)-l_T(q_T-l_T)\right] . \end{aligned}$$

We profit of the extra delta function $\delta (Q^2-m_T^2-f(l,q))$ to integrate over $\eta$. To find the result of this integration we should rewrite the function *f*(*l*, *q*) in terms of the variables of the problem

A.3 $$\begin{aligned} f(\eta ,l_T)&=2l_T \sqrt{Q^2+q_T^2} \cosh (\eta -y)-2l_T^2 \nonumber \\&\quad -2l_T \sqrt{q_T^2+l_T ^2-2 q_T l_T \cos \phi }, \end{aligned}$$

where $\phi$ is the angle between transverse momenta of lepton and *W* boson.

Thus, the delta function can be written as

A.4 $$\begin{aligned}&\delta (Q^2-m_T^2-f(l,q))= \frac{1}{2l_T \sqrt{Q^2+q_T^2}} \nonumber \\&\quad \times \delta \left[ \cosh (\eta -y)-\left( \frac{Q^2-m_T^2}{2l_T \sqrt{Q^2+q_T^2}} \right. \right. \nonumber \\&\qquad \left. \left. + \frac{l_T+\sqrt{q_T^2+l_T^2-2 q_T l_T \cos \phi }}{\sqrt{Q^2+q_T^2}}\right) \right] , \end{aligned}$$

giving a simple condition to perform the desired integral. Note that to do the integral we should rewrite the integral as

A.5 $$\begin{aligned} \frac{d^3l}{2E}&= \frac{dl^z}{2E} l_T dl_T d\phi = \frac{d\eta }{2} l_T dl_T d\phi \nonumber \\&=\frac{d \cosh (\eta -y)}{2\sqrt{\cosh ^2(\eta -y)-1}} l_T d l_T d\phi . \end{aligned}$$

Using the condition in Eq. (A.4) we can rewrite the delta function $\delta ((q-l)^2)$ of Eq. (A.2) to obtain a condition that allows to make the integral over $l_T$

A.6 $$\begin{aligned} \delta ((q-l)^2)&=\delta \left[ m_T^2-2l_T^2+2l_T q_T \cos \phi \right. \nonumber \\&\quad \left. -2 l_T \sqrt{l_T^2+q_T^2-2 q_T l_T \cos \phi }\right] . \end{aligned}$$

To rewrite this delta we should find the zeros of its argument. We find

A.7 $$\begin{aligned} l_T^{+}=&\frac{m_T^2}{2} \frac{q_T \cos \phi +\sqrt{m_T^2+q_T^2}}{m_T^2+q_T^2(1-\cos ^2 \phi )} >0, \end{aligned}$$

A.8 $$\begin{aligned} l_T^{-}=&\frac{m_T^2}{2} \frac{q_T \cos \phi -\sqrt{m_T^2+q_T^2}}{m_T^2+q_T^2(1-\cos ^2 \phi )} <0, \end{aligned}$$

so the solution $l_T^-$ is never used because we imposed that $l_T>0$. Thus the delta function over $l_T$ can be rewritten as

A.9 $$\begin{aligned} \delta ((q-l)^2)=A(Q^2,m_T^2,q_T,\cos \phi ) \delta (l_T-l_T^+), \end{aligned}$$

where

A.10 $$\begin{aligned}&A(Q^2,m_T^2,q_T,\cos \phi )\nonumber \\&\quad =\left| \frac{\sqrt{q_T^2+l_T^2-2 q_T l_T \cos \phi }}{2(l_T+\sqrt{q_T^2+l_T^2-2 q_T l_T \cos \phi })(l_T-q_T\cos \phi + \sqrt{q_T^2+l_T^2-2 q_T l_T \cos \phi })}\right| _{l_T=l_T^+} \end{aligned}$$

For simplicity we can rewrite the term coming from the Jacobian in Eq. (A.5) as

A.11 $$\begin{aligned} J(Q^2,q_T,\cos \phi )&=\frac{1}{\sqrt{\cosh ^2(\eta -y)-1}}\nonumber \\&=\left[ -1+\left( \frac{Q^2-m_T^2}{2l_T\sqrt{Q^2+q_T^2}}\right. \right. \nonumber \\&\quad \left. \left. +\frac{l_T+\sqrt{q_T^2+l_T^2-2 q_T l_T \cos \phi }}{\sqrt{Q^2+q_T^2}}\right) ^2\right] ^{-1/2}. \end{aligned}$$

Finally the term that only depends on momenta in Eq. (A.2) is rewritten as

A.12 $$\begin{aligned}&M(Q^2,m_T^2,q_T,\cos \phi )\nonumber \\&\quad =l(q-l)-l_T(q_T-l_T)\nonumber \\&\quad =\frac{Q^2-m_T^2}{2}+2l_T^2+l_T \sqrt{q_T^2+l_T^2-2 q_T l_T \cos \phi }\nonumber \\&\quad -2q_T l_T \cos \phi . \end{aligned}$$

Thus, the final result is written in term of the auxiliary functions *J*, *A*, *M* as a one-dimensional integral

A.13 $$\begin{aligned}&I_W(Q^2,m_T^2,q_T)\nonumber \\&\quad =\int _0^{2\pi } d\phi \frac{1}{4l_T\sqrt{Q^2+q_T^2}}\nonumber \\&\qquad \times J(Q^2,q_T,\cos \phi ) A(Q^2,m_T^2,q_T,\cos \phi )\nonumber \\&\qquad \times M(Q^2,m_T^2,q_T,\cos \phi )\theta (\mathrm{cuts}), \end{aligned}$$

that should be done numerically.

## Inputs from fits

In this section we report some results from previous fit [35] that we have used in this paper. The different PDF sets that have been reported in that fit and that we have used are listed in Table 4. The results for the TMD constants are in Table 5.

**Table 4:** List of collinear PDF used as the boundary for unpolarized TMDPDF

Short name	Full name	Ref.	LHAPDF id.
NNPDF31	NNPDF31_nnlo_as_0118	[28]	303600
HERA20	HERAPDF20_NNLO_VAR	[100]	61230
MMHT14	MMHT2014nnlo68cl	[27]	25300
CT14	CT14nnlo	[106]	13000
PDF4LHC	PDF4LHC15_nnlo_100	[107]	91700

**Table 5:** Values of $\chi ^2$ and NP parameters obtained in the fit of DY set of the data with different PDF inputs. Each set of PDF provide the corresponding value of $\alpha _s(M_Z)$

PDF set	$\chi ^2/N_{pt}$	Parameters for ${\mathcal {D}}$	Parameters for $f_1$	
HERA20	0.97	$B_\text {NP}=2.29\pm 0.43$	$\lambda _1=0.324\pm 0.029$	$\lambda _3=(3.56\pm 1.59)\times 10^2$
		$c_0=(2.22\pm 0.93)\times 10^{-2}$	$\lambda _2=13.2\pm 2.9$	$\lambda _4=2.05\pm 0.26$
				$$\lambda _5=-10.4\pm 3.5$$
NNPDF31	1.14	$B_\text {NP}=1.86\pm 0.30$	$\lambda _1=0.253\pm 0.032$	$\lambda _3=(3.47\pm 1.16)\times 10^2$
		$c_0=(2.96\pm 1.04)\times 10^{-2}$	$\lambda _2=9.0\pm 3.0$	$\lambda _4=2.48\pm 0.15$
				$$\lambda _5=-5.7\pm 3.4$$
MMHT14	1.34	$B_\text {NP}=1.55\pm 0.29$	$\lambda _1=0.198\pm 0.040$	$\lambda _3=(26.8\pm 13.2)\times 10^3$
		$c_0=(4.70\pm 1.77)\times 10^{-2}$	$\lambda _2=26.4\pm 4.9$	$\lambda _4=3.01\pm 0.17$
				$$\lambda _5=-23.4\pm 5.4$$
PDF4LHC	1.53	$B_\text {NP}=1.93\pm 0.47$	$\lambda _1=0.218\pm 0.041$	$\lambda _3=(9.26\pm 8.38)\times 10^2$
		$c_0=(3.66\pm 2.09)\times 10^{-2}$	$\lambda _2=17.9\pm 4.5$	$\lambda _4=2.54\pm 0.17$
				$$\lambda _5=-15.5\pm 4.7$$
CT14	1.59	$B_\text {NP}=2.35\pm 0.61$	$\lambda _1=0.277\pm 0.029$	$\lambda _3=(12.4\pm 3.2)\times 10^3$
		$c_0=(2.27\pm 1.33)\times 10^{-2}$	$\lambda _2=24.9\pm 2.9$	$\lambda _4=2.67\pm 0.13$
				$$\lambda _5=-23.8\pm 2.9$$

**Table 6:** Intervals for the TMD non-perturbative parameters calculated as explained in the text

$B_{NP}$	$c_0$	$\lambda _1$	$\lambda _2$	$\lambda _3$	$\lambda _4$	$\lambda _5$
2.01±0.19	$\left( \text {3.42}\pm \text {0.70}\right) \times \text {10}^{\text {-2}}$	0.248±0.015	18.3±1.5	$\left( \text {8.2}\pm \text {2.7}\right) \times \text {10}^{\text {2}}$	2.484±0.085	$-15.6\pm 1.7$

## Errors propagation from TMD parameters

An alternative way to present the errors coming from non-perturbative parametrization of the TMD is the following. We take from [35] the different values for the non-perturbative parameters coming from fits using several PDF sets. We have a data array of seven non-pertubative parameters and five values for each one with its respective uncertainty as it is shown in Table 5. We calculate the mean value of each parameter and its uncertainty associated doing the square sum of the uncertainties and taking the square-root of the sum. We show the values in Table 6. In order to quantify the uncertainty in our cross section due to the uncertainties in our non-pertubative parameters we use Eq. (C.1)

C.1 $$\begin{aligned} \begin{aligned} \Delta \left( \dfrac{d\sigma }{dq_T}\right)&=\sqrt{\sum _{ij}\dfrac{\partial }{\partial \theta _i}\left( \dfrac{d\sigma }{dq_T}\right) \bigg |_{\theta _i={\bar{\theta }}_i} \dfrac{\partial }{\partial \theta _j}\left( \dfrac{d\sigma }{dq_T}\right) \bigg |_{\theta _j={\bar{\theta }}_j}V_{ij}}\\&\simeq \dfrac{1}{2}\sqrt{\sum _{ij}\delta \left( \dfrac{d\sigma }{dq_T}\right) _i \delta \left( \dfrac{d\sigma }{dq_T}\right) _jC_{ij}}, \end{aligned} \end{aligned}$$

where $\theta _i=\{\text {B}_{\text {NP}},\text {c}_0,\lambda _1,\lambda _2,\lambda _3,\lambda _4,\lambda _5\}$ are the non-perturbative parameters and ${\bar{\theta }}_i$ their mean values collected in Table 5; $V_{ij}$ and $C_{ij}$ are the covariance and correlation matrices respectively of our non-perturbative parameters and $\delta \left( \frac{d\sigma }{dq_T}\right) _i$ is the difference of the differential cross section evaluated in the extremes of the interval of the non-perturbative parameter *i* and keeping the rest of non-perturbative parameters fixed at their mean value. In order to go from the first line to the second line, we have approximated the derivative of the cross section with respect to $\theta _i$ by

C.2 $$\begin{aligned}&\dfrac{\partial }{\partial \theta _i}\left( \dfrac{d\sigma }{dq_T}\right) \bigg |_{\theta _i={\bar{\theta }}_i}\nonumber \\&\quad \simeq \dfrac{1}{2\delta {\bar{\theta }}_i}\left( \dfrac{d\sigma }{dq_T} \left( {\bar{\theta }}_i+\delta {\bar{\theta }}_i,{\bar{\theta }}_{j\ne i}\right) -\dfrac{d\sigma }{dq_T}\left( {\bar{\theta }}_i-\delta {\bar{\theta }}_i, {\bar{\theta }}_{j\ne i}\right) \right) ,\nonumber \\ \end{aligned}$$

where $\delta {\bar{\theta }}_i$ is the uncertainty associated to the mean value of the non-perturbative parameter $\theta _i$. Using the relation between the correlation and covariance matrices $C_{ij}=\frac{V_{ij}}{\delta {\bar{\theta }}_i\delta {\bar{\theta }}_j}$ we arrive to Eq. (C.1).

The correlation matrix at NNLO for the $\lambda _i$ is provided in [35] and, as a reference, we have considered the central PDF of NNPDF31_nnlo_as_0118 [28].

The result of this error estimate is very similar to the one described in the main part of the paper, which is a confirmation of the Gaussian distribution of TMD parameter errors for this case.

## Footnotes

1 As usual NLO stands for next-to-leading order, NNLO for next-to-next-to-leading order, and so on.

2 We thank Pier Monni for communicating us that the AZ tune as provided by the Pythia collaboration does not coincide exactly with the one used in ATLAS experiment and used actually in [60] and one as provided by ATLAS experiment. We have included both in our plots which are shown in Figs. 9, 10, 11. The pictures show the differential cross section normalized to their integration over the shown interval. The errors from Pythia come from statistical uncertainty and we have checked that they are similar to the one obtained by the variation of the parameters of the tune.
